# Improvement of whole-cell transamination with *Saccharomyces cerevisiae* using metabolic engineering and cell pre-adaptation

**DOI:** 10.1186/s12934-016-0615-3

**Published:** 2017-01-03

**Authors:** Nora Weber, Marie Gorwa-Grauslund, Magnus Carlquist

**Affiliations:** 1 Division of Applied Microbiology, Department of Chemistry, Faculty of Engineering, Lund University, PO Box 124, 221 00 Lund, Sweden; 2 Evolva SA, Duggingerstrasse 23, 4153 Reinach, Switzerland

**Keywords:** Chiral amine, Whole-cell bioconversion, Amine transaminase, Pyridoxal-5′-phosphate, Pyruvate decarboxylase, Yeast, Co-substrate

## Abstract

**Background:**

Whole-cell biocatalysis based on metabolically active baker’s yeast with engineered transamination activity can be used to generate molecules carrying a chiral amine moiety. A prerequisite is though to express efficient *ω*-transaminases and to reach sufficient intracellular precursor levels.

**Results:**

Herein, the efficiency of three different *ω*-transaminases originating from *Capsicum chinense*, *Chromobacterium violaceum*, and *Ochrobactrum anthropi* was compared for whole-cell catalyzed kinetic resolution of *racemic* 1-phenylethylamine to (*R*)-1-phenylethylamine. The gene from the most promising candidate, *C*. *violaceum ω*-transaminase (CV-TA), was expressed in a strain lacking pyruvate decarboxylase activity, which thereby accumulate the co-substrate pyruvate during glucose assimilation. However, the conversion increased only slightly under the applied reaction conditions. In parallel, the effect of increasing the intracellular pyridoxal-5′-phosphate (PLP) level by omission of thiamine during cultivation was investigated. It was found that without thiamine, PLP supplementation was redundant to keep high in vivo transamination activity. Furthermore, higher reaction rates were achieved using a strain containing several copies of CV-TA gene, highlighting the necessity to also increase the intracellular transaminase level. At last, this strain was also investigated for asymmetric whole-cell bioconversion of acetophenone to (*S*)-1-phenylethylamine using l-alanine as amine donor. Although functionality could be demonstrated, the activity was extremely low indicating that the native co-product removal system was unable to drive the reaction towards the amine under the applied reaction conditions.

**Conclusions:**

Altogether, our results demonstrate that (*R*)-1-phenylethylamine with >99% *ee* can be obtained via kinetic resolution at concentrations above 25 mM *racemic* substrate with glucose as sole co-substrate when combining appropriate genetic and process engineering approaches. Furthermore, the engineered yeast strain with highest transaminase activity was also shown to be operational as whole-cell catalyst for the production of (*S*)-1-phenylethylamine via asymmetric transamination of acetophenone, albeit with very low conversion.

**Electronic supplementary material:**

The online version of this article (doi:10.1186/s12934-016-0615-3) contains supplementary material, which is available to authorized users.

## Background

Chiral amines are prevalent functional groups in a wide range of bioactive compounds, so efficient, sustainable, and economically feasible methods for their synthesis are highly desirable [1, 2]. In lieu of chemical catalysis, the use of *ω*-transaminase (*ω*-TA) (E.C. 2.6.1.18) has emerged as a competitive alternative for bio-catalysed production of chiral amines, either via asymmetric transamination of ketones or via kinetic resolution of *racemic* amines. Asymmetric transamination of prochiral ketones to chiral amines can be more advantageous than kinetic resolution of *racemic* substrates since in theory all substrate can be converted to the product as opposed to a maximum yield of 50% for kinetic resolution. Direct conversion of ketones to amines by transamination however often suffer from an unfavourable thermodynamic reaction equilibrium, which makes the reaction reliant on a functional co-product removal system [3]. Biocatalytic transamination is most often performed with purified enzymes or with cell extracts from recombinant bacteria over-expressing the required enzymes [3–5]. Processes where *ω*-TAs are used include for instance the synthesis of Sitagliptin, (*S*)-Rivastigmine, and precursors for the synthesis of Pregabalin and Brivaracetam [6–8].

From a process perspective, the use of intact whole microbial cells that over-express specific recombinant *ω*-TAs may be advantageous, since it offers a more simple overall process configuration with a reduced number of upstream unit operations and less generation of waste material [9, 10]. Additionally, cell metabolism can be exploited for the (re)-generation of co-factors and co- substrates provided that there is an attendant assimilation of a carbon and energy source during the reaction [11]. So far however there are no reported whole-yeast cell systems expressing recombinant *ω*-TAs that are operational in the direction from carbonyl to amine. For kinetic resolution on the other hand, recombinant yeast have previously been shown to be active in an aqueous buffer system supplemented with glucose as co-substrate [12]. Yet the whole-cell system demonstrated low specific activity, resulting in very low productivity and yield in the whole-cell biotransformation process. Further improvements of the whole-cell biocatalyst were achieved by coupling the transamination to a KRED (ketone reductase)-catalysed reduction to remove the co-product and thereby relieve product inhibition [13, 14]. Still, the catalytic activity of currently reported engineered yeast strains needs to be improved manifold in order to be competitive with current state-of-the art for *ω*-TA catalysis [3].

A potential limitation for kinetic resolution of *racemic* amines with metabolically active yeast cells is the availability of amine acceptors provided from glucose. *ω*-TAs of both bacterial [15–17] and plant [18–20] origin are able to utilize pyruvate as amine acceptor. Together with the central position of pyruvate in the carbon metabolism makes this a promising potential target to engineer for the improvement of whole-cell transamination. Elevated production of pyruvate in *Saccharomyces cerevisiae* was previously achieved by metabolic engineering of the pyruvate node by deletion of the pyruvate decarboxylase (*PDC*) genes *PDC1*, *PDC5*, and *PDC6* [21, 22]. The Pdc negative strain accumulated pyruvate, but was unable to grow on glucose without an external source of C_2_ compounds for synthesis of acetyl-CoA. Directed evolution of the Pdc negative strain in a long-term continuous cultivation setup led to the phenotypic trait of growth at high glucose concentration and pyruvate accumulation in aerobic batch mode without the necessity to add acetate [23]. It can be speculated that this strain would be an efficient platform for resolution of *racemic* amines by whole-cell transamination.

Another engineering target that was previously shown to influence whole-cell transamination activity is the concentration of pyridoxal-5′-phosphate (PLP) [12, 24], which is an essential co-factor for all aminotransferases. To increase the activity of PLP-dependent reactions, PLP is typically supplemented directly to the reaction solution thereby relieving potential limitations in availability [12, 25, 26]. An alternative to supplementation is the increase of intracellular levels by metabolic engineering of PLP biosynthetic pathways. PLP is synthetized in two pathways: the ribose 5-phosphate-dependent de novo pathway [27–31], and the PLP-salvage pathway where the pyridoxine, pyridoxal, and pyridoxamine scaffolds are interconverted and phosphorylated at the 5′-hydroxyl group [32, 33]. It was recently shown that by introducing *PdxS* and *PdxT* encoding a PLP synthase complex originating from *Bacillus subtilis* into *Escherichia coli*, the intracellular PLP levels increased 2.4-fold [24]. This resulted in similar specific whole-cell activity for biotransformation of l-lysine to cadaverine by a PLP-dependent lysine decarboxylase (*CadA*), with or without the addition of PLP in the reaction solution [24]. In yeast, PLP biosynthesis has been found to correlate to extracellular concentration of thiamine, which is an essential vitamin for cell growth and is typically part of defined mineral media [34]. Regulation of de novo biosynthesis of PLP and thiamine are thus closely intertwined, and the biosynthetic activity required for their formation is inversely correlated with extracellular availability [35, 36]. Based on this knowledge, we hypothesize that the omission of thiamine in the cultivation medium that lead to an increased intracellular PLP concentration will result in an improvement in specific whole-cell activity.

**Fig. 1:**
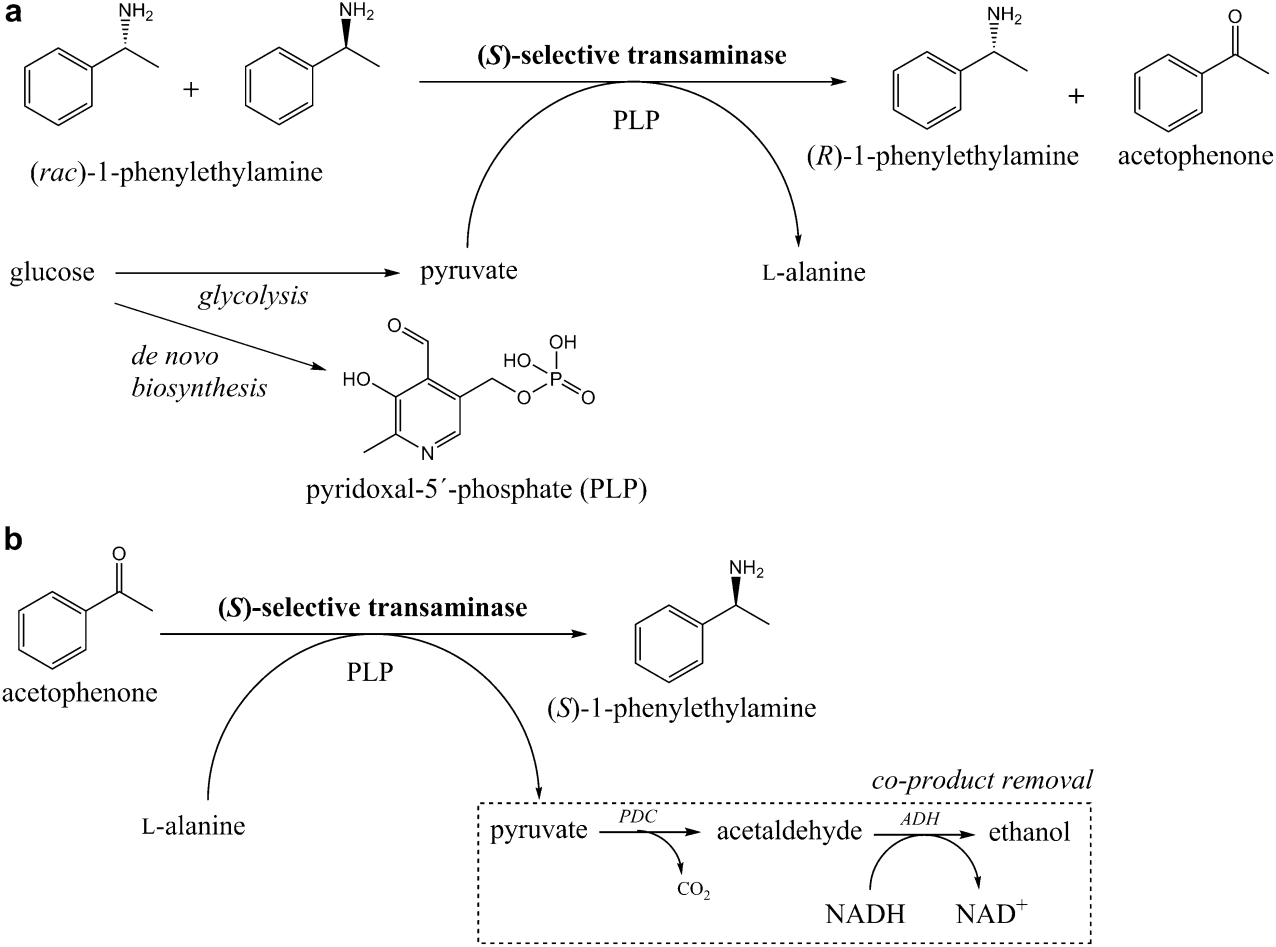
**a** Whole-cell kinetic resolution of *racemic* 1-PEA with (*S*)-selective ω-transaminase to (*R*)-1-PEA and acetophenone. The mechanism for enzymatic transamination consists of two half-reactions under which the co-factor PLP is regenerated, and the amine acceptor/donor is converted into the corresponding amine/ketone. PLP and the amine acceptor pyruvate is formed in the cell by concomitant conversion of glucose. **b** Whole-cell asymmetric conversion of acetophenone to (*S*)-1-PEA by (*S*)-selective ω-transaminase. The co-product pyruvate is removed from the system by further conversion catalysed by PDC and ADH resulting in formation of CO_2_ and ethanol

In the present study, we investigated different bioengineering strategies to modify the intracellular environment of *S. cerevisiae* with the aim to increase specific in vivo transamination activity. As model reaction, we used kinetic resolution of *racemic* 1-phenylethylamine (PEA) to (*R*)-1-PEA with sole addition of glucose to supply PLP and pyruvate as amine acceptor (Fig. 1a). First, the specific in vivo transaminase activity of three *ω*-TAs with the same substrate and stereo-selectivity, but with distinct origin (plant and bacteria), different pH optima, and reaction kinetics were compared. Differences in specific in vivo activities were observed, which sheds light into potential desirable enzyme characteristics for optimal transamination with intact yeast cells. Subsequently, a previously developed Pdc negative pyruvate accumulating strain [23] was evaluated for its potential as platform host for whole-cell kinetic resolution. Furthermore, we analysed the effect of thiamine omission in the cultivation medium with the aim to increase intracellular PLP levels and thereby decrease the dependence of adding PLP to the reaction solution. We also increased the TA gene copy number sixfold, which in combination with cultivation in thiamine-free medium and with an increased biomass concentration during the bioconversion resulted in complete kinetic resolution of 25 mM *racemic* 1-PEA to (*R*)-1-PEA in a reaction configuration with glucose as co-substrate. Finally, the most active yeast catalyst was investigated for direct asymmetric transamination of acetophenone to (*S*)-1-PEA with l-alanine as amine donor (Fig. 1b). We speculated that the endogenous pyruvate dissimilating pathways, e.g. conversion of pyruvate to acetaldehyde and CO_2_ by Pdc, would function as an efficient co-product removal system. We could indeed confirm that the desired reaction was operational by whole-cell transamination, however, at extremely low conversion.

## Results

### Comparison of three different *ω*-transaminases for whole-cell transamination in *S. cerevisiae*

**Fig. 2:**
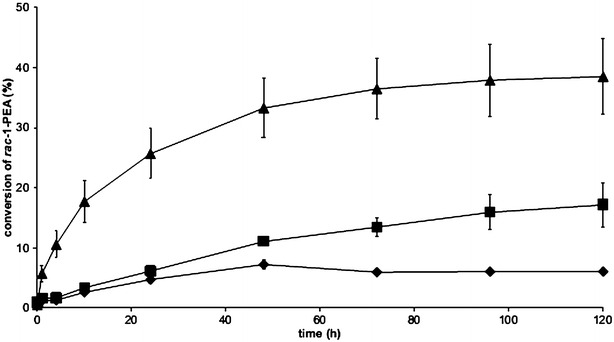
Kinetic resolution of *racemic* 1-phenylethylamine (PEA) with whole cells of TMB4367 (CC-TA) (*diamond*), TMB4369 (CV-TA) (*triangle*), and TMB4371 (OA-TA) (*square*) *S. cerevisiae* strains. Experiments were performed at least in biological duplicates using 100 g/l glucose, 25 mM *racemic* 1-PEA, 0.1 mM PLP, and 5 g/l dw cells. The conversion (%) refers to *racemic* 1-PEA, with a theoretical maximum of 50%. No error bar is visible if the standard deviation lies below 5%

*S. cerevisiae* expressing *ω*-TA gene from the chili pepper plant *C. chinense* (CC-TA) was previously shown to be functional as whole-cell biocatalyst for the kinetic resolution of *racemic* 1-phenylethylamine (PEA) to (*R*)-1-PEA, but with relatively low specific activity [12]. To investigate if there was a more suitable transaminase for the yeast bio-catalytic system, two additional *S. cerevisiae* strains were constructed containing two other TAs: the well-characterized *ω*-TA from the beta-proteobacterium *C. violaceum* (CV-TA) [15, 37, 38] and the previously reported *ω*-TA from the alpha-proteobacterium *O. anthropi* (OA-TA) [17, 42], which has shown neither substrate nor product inhibition. The three enzymes all possess similar substrate specificity and enantio-selectivity; however, they have differences in acetophenone inhibition kinetics and optimal pHs (Table 1). In the present study, the codon-optimized synthetic genes were cloned into a yeast integrative vector (YIpNW) (Table 2) and transformed into the parental strain TMB4150 generating CV-TA strain (TMB4369) and OA-TA strain (TMB4371) (Table 3). The three strains were compared for the kinetic resolution of *racemic* 1-PEA in a reaction configuration where PLP was supplemented and glucose was used as co-substrate for the supply of amine acceptors. As previously observed [12], CC-TA strain converted (*S*)-1-PEA to acetophenone (ACP) during 48 h after which the reaction stopped, reaching only 6.1% final conversion (Fig. 2). The reaction rate of OA-TA strain was similar to CC-TA strain during the first 4 h (0.16 mmol (*S*)-1-PEA/g dw/h vs. 0.15 mmol (*S*)-1-PEA/g dw/h, respectively), however, the OA-TA strain continuously resolved *racemic* 1-PEA until 120 h, thereby achieving almost threefold higher conversion (17.2%). The best result was obtained with CV-TA strain that had a more than fourfold higher initial specific activity (0.64 mmol (*S*)-1-PEA/g dw/h) than the CC-TA strain. Although the reaction rate decreased between 4 and 120 h, 38.5% conversion was achieved after 120 h, resulting in the highest *ee* (64.4%) of (*R*)-1-PEA.

**Table 1:** ω-Transaminases under investigation in the present study

ω-Transaminase	Species origin of the enzyme	Inhibited by acetophenone	Enantio-selectivity	pH_opt_	References
CC-TA	*Capsicum chinense*	*Yes*	*S*	7–8	[18]
CV-TA	*Chromobacterium violaceum*	*Yes*	*S*	8.5–9	[15, 37, 38]
OA-TA	*Ochrobactrum anthrophi*	*No*	*S*	9	[17, 42]

**Table 2:** Plasmids used in the present study

Plasmids	Description	References
YIpOB7	TDH3 promoter, ADH1 terminator, *XDH* under PGK1 promoter, with PGK1 terminator, *URA3* gene, *AMP* resistance gene	[54]
YIpNW	YIpOB7 without XDH, TDH3 promoter, ADH1 terminator, PGK1 promoter, PGK1 terminator, *URA3* gene, *AMP* resistance gene	This study
pNW10	*CC*-*TA* ORF cloned in YIpNW between TDH3 promoter and ADH1 terminator, *URA3* gene, *AMP* resistance gene	[12]
pUC57-CV	Codon-optimized *CV*-*TA* gene	GenScript, NJ, USA
pUC57-OA	Codon-optimized *OA*-*TA* gene	GenScript, NJ, USA
pNW12	*CV*-*TA* ORF between TDH3 promoter and ADH1 terminator, *URA3* gene, *AMP* resistance gene	This study
pNW14	*OA*-*TA* ORF between TDH3 promoter and ADH1 terminator, *URA3* gene, *AMP* resistance gene	This study

**Table 3:** *S. cerevisiae* strains used in the present study

Strains	Description	References
TMB4150	*CEN.PK2*-*1C MAT* **a** *ura3*-*52 MAL2*-*8* ^*C*^ *SUC2, TRP1, LEU2, HIS3,* auxotrophies *trp1, leu2* and *his3* were reversed by transformation with PCR-amplified intact genes	Jan Knudsen, unpublished
TMB4367	TMB4150 containing pNW10, overexpressing *CC*-*TA* encoding gene, *TRP1*, *LEU2*, *HIS3*	[12]
TMB4369	TMB4150 containing pNW12, overexpressing *CV*-*TA* encoding gene, *TRP1*, *LEU2*, *HIS3*	This study
TMB4371	TMB4150 containing pNW14, overexpressing *OA*-*TA* encoding gene, *TRP1*, *LEU2*, *HIS3*	This study
TAM	MAT**a** *pdc1*(-6,-2)::*loxP pdc5*(-6,-2)::*loxP pdc6*(-6,-2)::*loxP ura3*-*52*, selected for C_2_ independence in glucose-limited chemostats and glucose-tolerant growth in batch culture	[23]
TMB4374	TAM containing pNW12, overexpressing *CV*-*TA* encoding gene, *Δpdc1,5,6*	This study
TMB4375	TMB4150 containing pNW12 (6 copies), overexpressing *CV*-*TA* encoding gene, *TRP1*, *LEU2*, *HIS3*	This study

### Influence of intracellular pyruvate accumulation on whole-cell transamination

The intracellular pyruvate derived from glucose through glycolysis may be limited for transamination due to the presence of endogenous pyruvate-catalysing enzymes such as the pyruvate dehydrogenase complex (Pdh), pyruvate decarboxylase (Pdc), and pyruvate carboxylase (Pyc) [39]. In order to verify this hypothesis, whole-cell transamination was performed using the pyruvate-accumulating TAM strain [23]. As CV-TA had the best properties for whole-cell transamination, the TAM strain was transformed with plasmid pNW12 resulting in CV-TA TAM strain (TMB4374) (Tables 2, 3). To prepare biomass for the subsequent whole-cell reaction, CV-TA and CV-TA TAM strains were grown in a mineral medium containing 20 g/l glucose as well as 3 g/l ethanol to increase the growth rate of CV-TA TAM strain as previously reported [40]. Under these conditions, the latter reached a final OD_620_ of 5.3 ± 0.2 after 31 h, compared to OD_620_ 7.1 ± 0.1 after 24 h for CV-TA strain (Additional file 1: Figure S1). Glucose consumption was nearly sixfold faster for CV-TA strain with ethanol as primary by-product, compared to CV-TA TAM strain that did not produce ethanol. Under the evaluated conditions, both strains produced pyruvate concentrations below the detection limit.

**Fig. 3:**
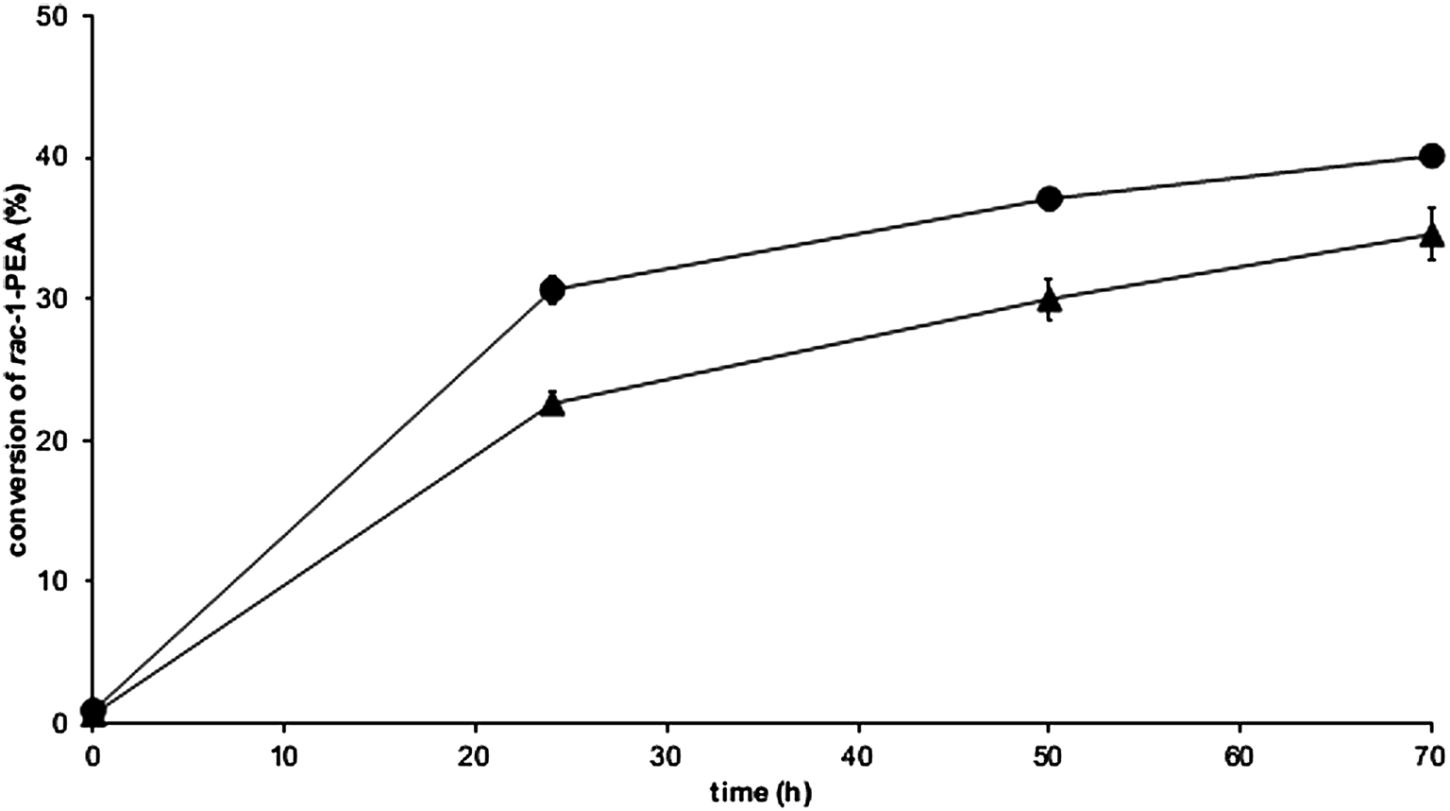
Comparison of TMB4369 (CV-TA) (*triangle*) and TMB4374 (CV-TA TAM) strain (*circle*) for the kinetic resolution of *racemic* 1-PEA using whole cells. The experiments were performed in biological duplicates with 100 g/l glucose, 25 mM *racemic* 1-PEA, 0.1 mM PLP, and 5 g/l dw cells. No error bar is visible if the standard deviation lies below 5%

**Fig. 4:**
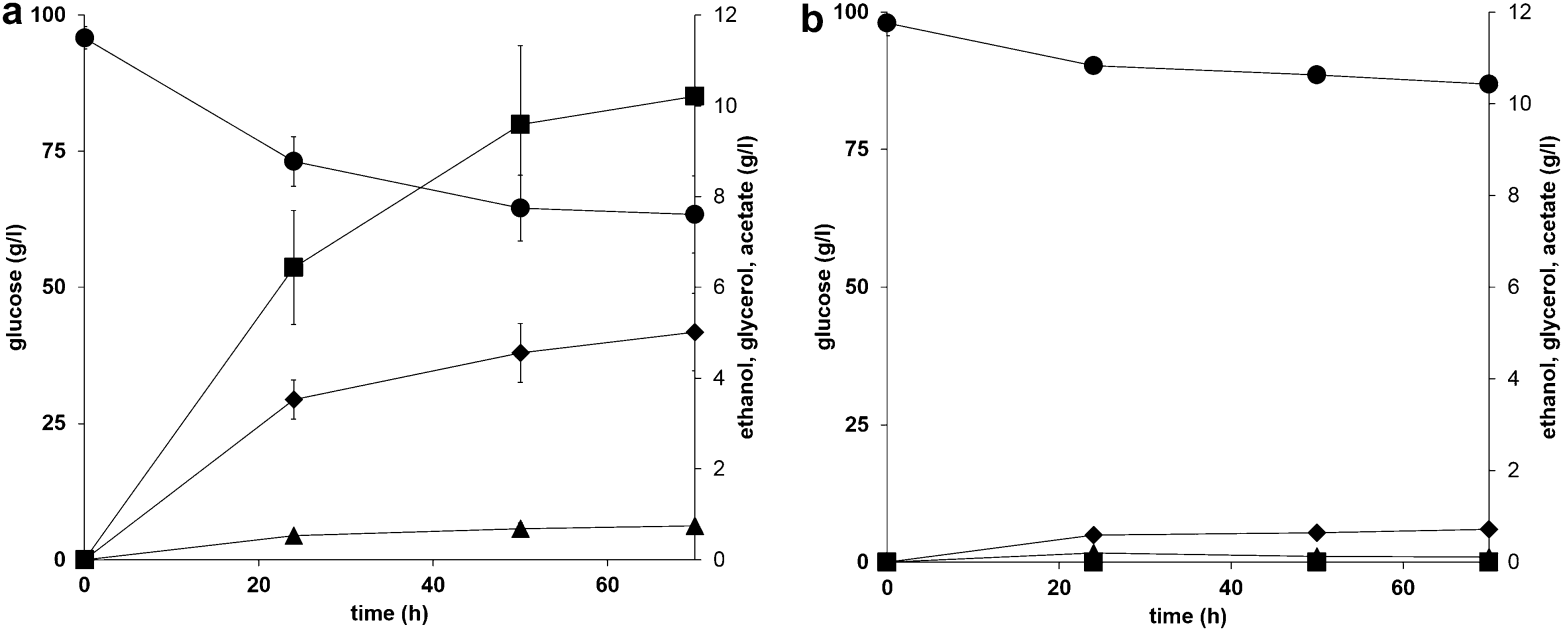
Metabolites during kinetic resolution of *racemic* 1-PEA with **a** TMB4369 (CV-TA) and **b** TMB4374 (CV-TA TAM) strain. Glucose (*circle*), glycerol (*diamond*), acetate (*triangle*), ethanol (*square*). No error bar is visible if the standard deviation lies below 5%

When investigated for whole-cell transamination, the CV-TA strain showed 34.6% conversion after 70 h (Fig. 3), while the CV-TA TAM strain had slightly higher conversion (40.1% conversion after 70 h). The conversion for the CV-TA strain was the same as for the experiments described above (Fig. 2), thereby demonstrating that supplementation of ethanol in the medium during the pre-cultivation step did not influence the specific activity of the whole-cell biocatalyst. Glucose consumption and formation of ethanol and glycerol during the progress of the reaction were significantly lower for the CV-TA TAM strain as compared to the control (Fig. 4a, b). Thus, the amount of glucose required to provide sufficient levels of amine acceptor was lower in the TAM strain background. Extracellular pyruvate accumulation was not detected for either of the strains, which in the case of the TAM strain is most probably due to a combination of depletion by the transaminase-catalysed reaction and an insufficient aeration under the applied reaction conditions.

### Omission of thiamine in the cultivation medium during biocatalyst production improves whole-cell biocatalyst activity

Availability of intracellular PLP contributes to a significant degree to the activity of PLP-dependent enzymes in living cells, as has been observed previously [12]. We therefore investigated whether the omission of thiamine in the cultivation medium, which previously was shown to result in increased PLP levels, would also result in higher specific whole-cell transamination activity in a subsequent bioconversion step. When cultivated in a medium without thiamine, the CV-TA strain (TMB4369) displayed a substantially reduced maximal specific growth rate (0.24 ± 0.0/h) as compared to when thiamine was added (0.31 ± 0.0/h), but no substantial effect on the by-product distribution (ethanol, glycerol, acetate) was observed. Electrospray Ionization Mass Spectrometry (ESI–MS) analysis was used for measurement of intracellular PLP, and it could indeed be demonstrated that the omission of thiamine led to a 1.5- to 2-fold increase in concentration (data not shown).

**Fig. 5:**
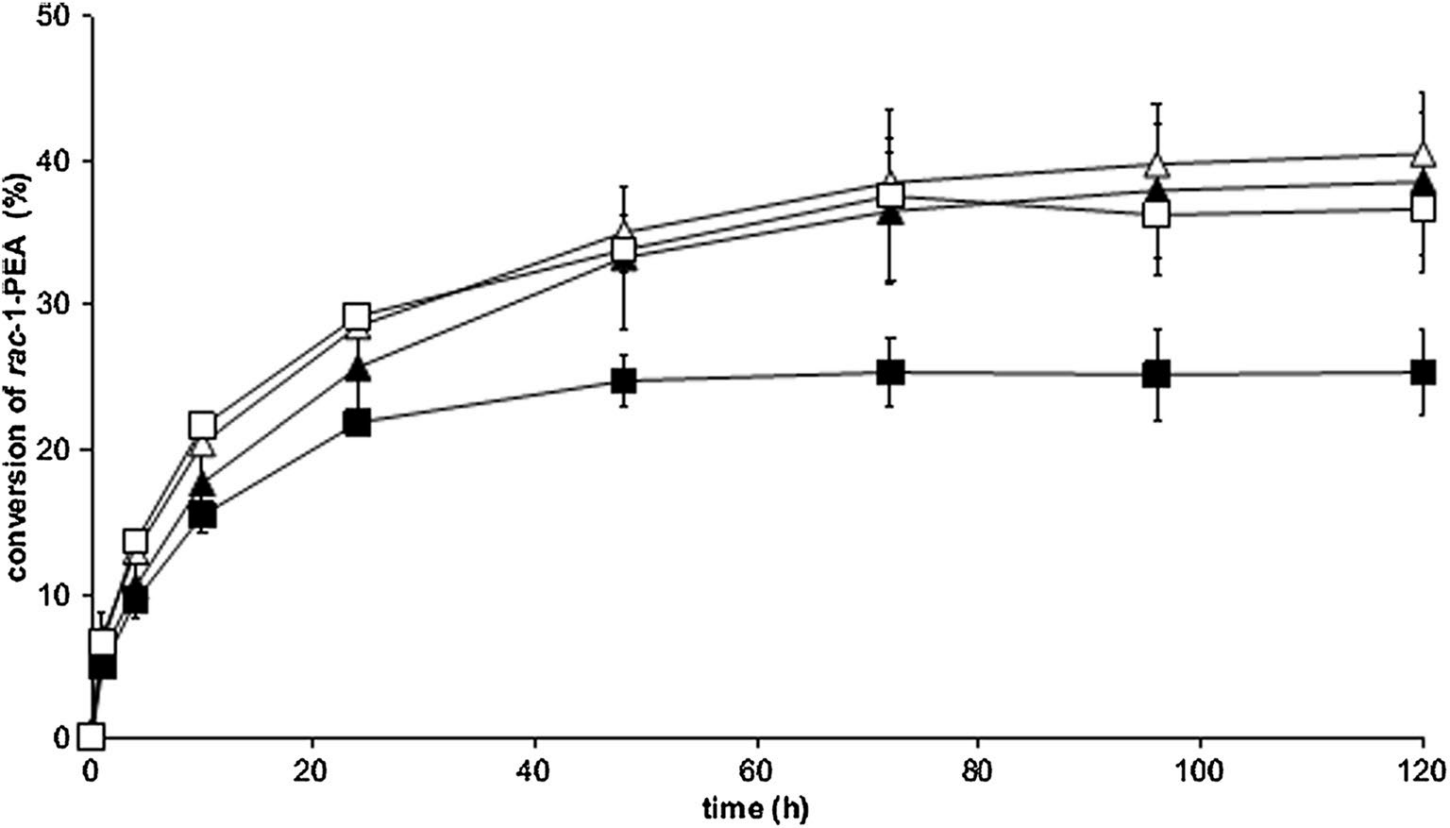
Kinetic resolution of *racemic* 1-PEA with TMB4369 (CV-TA) with and without addition of PLP to the reaction solution. Prior to the reaction cells were pre-cultivated with or without thiamine in the medium. Experiments were performed in biological triplicates using 100 g/l glucose, 25 mM *racemic* 1-PEA, and 5 g/l dw cells. Symbols: *empty square* (0 mM PLP in the reaction, 0 µM thiamine in culture medium); *empty triangle* (0.1 mM PLP in the reaction, 0 µM thiamine in culture medium); *filled square* (0 mM PLP in the reaction, 3.3 µM thiamine in culture medium); *filled triangle* (0.1 mM PLP in the reaction, 3.3 µM thiamine in culture medium). No error bar is visible if the standard deviation lies below 5%

To see the effect of thiamine on whole-cell bioconversion, the yeast cells were used for transamination with and without further addition of PLP. When thiamine was present in the cultivation medium, a substantially higher conversion was obtained with PLP added to the reaction solution (38.5% as compared to 25.4% without addition of thiamine; Fig. 5). In contrast, when thiamine was omitted in the culture step, the addition of PLP in the biotransformation step had a smaller effect on the conversion, i.e. 40.5% conversion was obtained compared to 36.7% conversion without the addition of PLP. Thus, it can be concluded that omission of thiamine during cultivation indeed increased the whole-cell activity in the subsequent reaction step.

### Influence of TA gene copy number and cell loading on whole-cell transamination

**Fig. 6:**
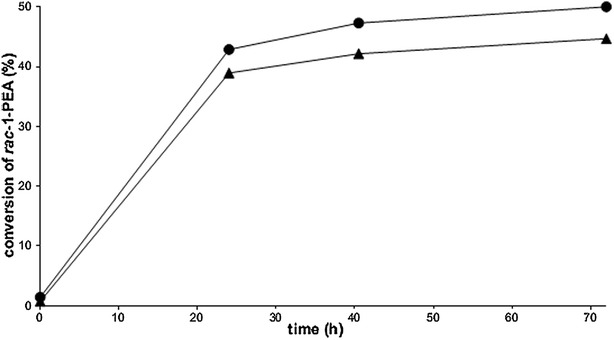
Kinetic resolution of *racemic* 1-PEA with TMB4375 (6× CV-TA). Experiments were performed in biological duplicates using 100 g/l glucose, 25 mM *racemic* 1-PEA, and 5 g/l dw (*triangle*) or 25 g/l dw (*circle*) cells. No error bar is visible if the standard deviation lies below 5%

It has previously been shown that an increased transaminase gene copy number led to higher specific in vivo transaminase activity [13]. To evaluate if this observation still held with a *ω*-TA having higher specific in vivo enzyme activity in yeast, a strain with sixfold higher number of copies of the CV-TA gene (TMB4375) was constructed and used for whole-cell transamination. The whole-cell biocatalyst was produced without adding thiamine in the medium during the cultivation step, and glucose was used as sole co-substrate in the subsequent reactions that were performed without addition of PLP to the solution. Indeed, when using the same amount of cells (5 gdw/l) for the reaction, a considerable higher conversion (44.7%) was reached (Fig. 6) compared to the strain carrying only onefold copies of CV-TA gene (36.7%) (Fig. 5).

To see if it would be possible to reach full conversion by increasing the amount of the whole-cell biocatalyst with high TA-gene copy-number, kinetic resolution of 25 mM *racemic* 1-PEA was carried out with two cell loadings (5 and 25 g/l dw). The initial reaction rate for both cell loadings was very similar (0.33 mmol (*S*)-1-PEA/g dw/h with 25 g/l dw vs. 0.32 mmol (*S*)-1-PEA/g dw/h with 5 g/l dw) during the first 24 h. However, with 25 g/l dw whole-cell biocatalyst, 50% conversion and an *ee* of (*R*)-1-PEA > 99% was reached within 72 h (Fig. 6).

### Whole-cell transamination of acetophenone to (*S*)-1-phenylethylamine

As an alternative to kinetic resolution of a *racemic* substrate, chiral amines can be produced by direct asymmetric transamination of the ketone. However, the reaction equilibrium for ω-transaminations often lies in the direction towards the ketone which is a major challenge for reaching high conversion. In the case for direct synthesis of (*S*)-1-PEA from acetophenone using amino acids (e.g. l-alanine) as amine donor, the Gibbs free energy was calculated using the group contribution method [41] to be ΔG_0_′ = +4.01 kcal/mol, i.e. the reaction is thus not thermodynamically favourable. It can be hypothesised that the native pyruvate dissimilating enzymes in yeast, e.g. Pdc, pyruvate dehydrogenase (Pdh), and pyruvate carboxylase (Pyc), in principle may be used for co-product removal and thereby drive the reaction towards the amine. To shed some light into the potential of the native pyruvate metabolism to drive asymmetric transamination, the conversion of acetophenone to (*S*)-1-PEA with addition of an excess of l-alanine was evaluated with the yeast strain expressing six copies of the CV-TA gene (TMB4375) and thereby having the highest transaminase activity. Indeed, the product was detected after bioconversion for 24 h, but at very low titers (0.35 mM (*S*)-1-PEA, Additional file 1: Figure S2), demonstrating that the reaction indeed is possible but significantly limited under the conditions used. In addition to (*S*)-1-PEA, the formation of low amount (*S*)-1-phenylethanol was also detected, demonstrating that endogenous acetophenone reductases are also active in the whole-cell system under the applied reaction conditions (Additional file 1: Figure S2).

## Discussion

In this study, major improvements in whole-cell transamination with engineered *S. cerevisiae* were achieved through the identification of relevant parameters, such as the type and intracellular level of *ω*-transaminase. Also, the need to increase the intracellular concentration of the involved co-substrates and co-factors was investigated. The use of a *S. cerevisiae* strain with elevated pyruvate concentration enabled slightly higher conversion than the reference strain. Furthermore, cells grown in the absence of thiamine gave higher conversion under conditions where PLP was omitted. Notably, complete kinetic resolution of 25 mM *racemic* 1-phenylethylamine was achieved with glucose as co-substrate and using a strain containing six copies of CV-TA, high cell density, and no added PLP. To the best of our knowledge, the yeast catalyst TMB4375 described herein thus has the highest *ω*-TA activity so far reported for a metabolically active whole-cell system that exploits cell metabolism to supply both PLP and amine acceptor for the reaction.

Enzymes that are optimized for purified systems might not be optimal for a whole-cell system due to factors such as incompatible pH optima, temperature optima, or enzyme kinetics. pH optima of 7–8, 9, and 8.5–9 were previously recorded for the *ω*-TA from *C. chinense* [18], *O. anthropi* [42] and *C. violaceum* [37], respectively, which should be compared with *S. cerevisiae* intracellular cytosolic pH of 7–7.5 (depending on extracellular pH, and growth phase) [43, 44]. Therefore, this parameter does not per se explain the highest conversion observed with CV-TA. Comparison of in vitro activities of the different enzymes does not clarify the better performance of CV-TA in the whole-cell system either, since CV-TA showed more than twofold higher conversion than CC-TA but nearly fourfold lower conversion than OA-TA in cell extracts (Additional file 1: Figure S3). In whole cells however, the intracellular level of substrate, co-substrates, and co-factors is highly dependent on cell metabolism and intermembrane transport, which contrasts with isolated enzyme catalysts where the levels of the different components can be freely adjusted. This leads to the observation that the enzyme and host metabolism need to be compatible and enzyme evaluation should be performed in combination with the host. It has been claimed that OA-TA has no substrate or product inhibition [17], and it indeed displayed minor reduction of the reaction rate during the whole-cell process compared to CV-TA and CC-TA. OA-TA has previously been reported to have a greater K_m_ value compared to other *ω*-TAs [17]. Therefore, we believe that the observed lower reaction rates for OA-TA in the whole-cell system was due to lower substrate affinity and suboptimal intracellular substrate concentration, as the substrate has to diffuse through the cell membrane.

In addition to the role of enzyme kinetics, we also highlighted the necessity to provide sufficient amount of the biocatalyst as full conversion was achieved by both increasing the TA gene copy number, i.e. the intracellular TA level, and to provide an increased amount of cells. It is likely that an even faster process can be achieved by introducing a reductase, which will further convert ACP to its less inhibitory alcohol product, as efficiently demonstrated previously by over-expressing a KRED from *Lactobacillus kefir* together with CC-TA in *S. cerevisiae* [13], or by the use of endogenous reductases to releave inhibition of recombinant *Vibrio fluvialis* JS17 ω-TA in *Pichia pastoris* [45].

We previously demonstrated that glucose could represent a significantly cheaper co-substrate than pyruvate for whole-cell transamination, by providing not only intracellular pyruvate through glycolysis, but also giving higher cell viability and higher conversion without the addition of co-factor PLP [12]. In order to limit pyruvate dissimilation through ethanol formation, the deletion of Pdc activity [23] was seen as the next step in process optimization as more pyruvate should be made available inside the cells. Previous trials using the Pdc negative strain indeed led to about 20 g/l extracellular pyruvate production after 24 h from 100 g/l glucose. During the cultivation of CV-TA TAM strain a significantly lower amount (1.5–2 g/l) pyruvate was measured. This may be explained by the drastic reduction of the pH (2.7 after 24 h) and limitation in aeration, which is needed for the re-oxidation of NADH. Despite of this, slightly higher conversion of *racemic* 1-phenylethylamine was achieved compared to the control strain with PDC activity.

Omission of thiamine in the culture medium used for production of the whole-cell biocatalyst led to substantially improved transamination in the absence of added PLP co-factor. Thiamine (vitamin B_1_) has previously been suggested to inhibit PLP (one form of vitamin B_6_) synthesis in *Saccharomyces carlsbergensis* 4228 [35]. Also a *S. cerevisiae* laboratory strain derived from S288C had a substantially lower specific growth rate when thiamine and no pyridoxine were added to the culture medium [46]. This was suspected to be due to a high affinity of *THI10* encoded thiamine transporter [47], which led to increasing intracellular thiamine concentration and repression of PLP biosynthesis. There is also a connection between thiamine and PLP on a biosynthetic level, as PLP, or one of its closely related forms, and histidine are building blocks for one of the precursors (4-amino-5-hydroxymethyl-2-methylpyrimidine monophosphate (HMP-P)) of thiamine [48, 49]. It has additionally been reported that genes *SNO2*, *SNO3*, *SNZ2,* and *SNZ3* are up-regulated in the absence of thiamine [36], and that overexpression of these genes in a laboratory strain leads to nearly the same specific growth rate when thiamine and not pyridoxine were added to the culture medium [46]. *SNO1* and *SNZ1*, which are genes coding for enzymes synthesizing PLP in the de novo pathway in *S. cerevisiae* [28, 31, 50], have a very high sequence similarity to *SNO2*, *SNO3* and *SNZ2*, *SNZ3* respectively. Here we demonstrated that thiamine deficiency indeed led to higher intracellular PLP concentration. Even if the maximal growth rate was reduced by 20%, the final cell density was in a similar range after 24 h (OD_620_ = 7.0 ± 0.4 with thiamine, OD_620_ = 6.6 ± 0.4 without thiamine). Overall, the omission of thiamine led to a higher conversion, and improved the process by two factors: first the addition of thiamine and PLP was avoided and second, higher conversion was achieved.

In conclusion, the best reaction set-up for kinetic resolution of *racemic* 1-phenylethylamine with glucose as only co-substrate consists of using the strain with several copies of CV-TA with high cell loading, and performing cultivation without thiamine. The use of metabolically active yeast over-expressing selective transaminases for biocatalytic transamination is a promising strategy, since it is simple and requires only glucose as co-substrate for the supply of both the amine acceptor and the co-factor PLP.

We report also the use of *S. cerevisiae* for asymmetric synthesis of (*S*)-1-PEA, albeit with low conversion. To combat the unfavourable reaction equilibrium that lies in direction of the ketone, co-product removal has previously been shown to improve reaction efficiency. For the use of l-alanine as amine donor, a multitude of enzymatic in vitro co-product removal systems have previously been developed, e.g. based on the conversion of pyruvate back to l-alanine by alanine dehydrogenase (Aldh) [26, 51], or pyruvate to acetaldehyde and CO_2_ by Pdc [52]. Yeast possess a number of enzymes that catalyse the conversion of pyruvate, e.g. into acetaldehyde by Pdc and further to ethanol by alcohol dehydrogenase. However, in our hands the engineered yeast catalyst was operational with only very low activity in the direction towards the amine, which indicates that the native pyruvate metabolism was either not functional efficiently or that it did not have the capacity to remove pyruvate under the tested conditions. It is likely that engineering of process conditions in combination with further increase of transaminase activity and of pyruvate dissimilatory pathways may improve conversion. An alternative strategy may be to explore other amine donors that have a more favourable thermodynamic equilibrium and/or more efficient co-product removal.

## Methods

### Chemicals

Acetophenone (ACP), *racemic* 1-phenylethylamine (1-PEA), (*R*)-1-PEA and (*S*)-1-PEA were bought from Merck (Hohenbrunn, Germany), pyridoxal-5′-phosphate (PLP) from AppliChem (Darmstadt, Germany), and all other chemicals from VWR (Leuven, Belgium).

### Strains, media, and cell growth

*Escherichia coli* strain DH5α (Life Technologies, Rockville, MD, USA) was used for subcloning. *S. cerevisiae* strain TMB4150 (*MAT* **a**, *ura3*-*52 MAL2*-*8* ^*C*^ *SUC2*) was kindly provided by Jan Knudsen, Applied Microbiology, Lund University, Sweden. TAM strain was kindly provided by Antonius van Maris, Department of Biotechnology, Delft University of Technology, Netherlands. *S. cerevisiae* strains TMB4367, TMB4369, TMB4371, TMB4374, and TMB4375 (see construction below; Tables 2, 3) were used for whole-cell transamination. Strains were kept as 20% glycerol stocks at −80 °C and grown on solid media for 2 days prior to experiments.

Transformation and cell growth was performed as described previously [12] except that defined mineral medium [53] was used instead of YPG medium. The mineral medium without thiamine contained the same concentration as previously described except for thiamine [53]. For cultivation of the pyruvate decarboxylase deletion mutant TAM, 3 g/l ethanol was added to the mineral medium.

### Nucleic acid manipulation

Plasmid DNA was prepared with the GeneJET Plasmid Miniprep Kit (Thermo Scientific, Rockford, IL, USA) and agarose gel DNA extraction was performed using QIAquick® Gel Extraction Kit (Qiagen GmbH, Hilden, Germany). Primers from MWG-Biotech AG (Ebersberg, Germany) and *Phusion* Hot Start II DNA Polymerase and dNTPs from Thermo Scientific (Rockford, IL, USA) were used for polymerase chain reactions (PCR) and performed in a GeneAmp PCR system 9700 (Applied Biosystems, Foster City, CA, USA). PCR products were purified with the GeneJET PCR Purification Kit (Thermo Scientific, Rockford, IL, USA). Sequencing was performed by MWG-Biotech AG (Ebersberg, Germany). InFusion® HD Cloning Kit (Clontech Laboratories, Mountain View, CA, USA) was used for DNA manipulation.

### Strain construction

The yeast-codon optimized coding regions of the *ω*-TA genes from *C. violaceum* (GenBank: WP011135573.1, Swiss-Prot: Q7NWG4) and from *O. anthropi* (GenBank: YP001368759.1, Swiss-Prot: A6WVC6) were PCR amplified from pUC57-CV and pUC57-OA (Table 2), respectively, using the primers listed in Additional file 1: Table S1. YIpOB7 was cut with *Bgl*II to remove XDH gene and self-ligated to create YIpNW, which was cut with *Xba*I and the PCR fragments inserted by InFusion® cloning, thus creating pNW12 and pNW14 (Table 2). Correct orientation of the inserts and sequences were verified by restriction enzyme analysis and sequencing. Integrative vectors pNW12 and pNW14 were cleaved with *Apa*I within the *URA3* marker gene and then used to transform the haploid laboratory strain TMB4150, resulting in CV-TA strain (TMB4369) and OA-TA strain (TMB4371), or the TAM strain, which resulted in CV-TA TAM strain (TMB4374) (Table 3). The 6× CV-TA strain (TMB4375) was constructed by transforming the haploid laboratory strain TMB4150 with pNW12 and screening a large number of transformants with qPCR for multiple single-crossing over integration events. Determination of relative gene copy number by qPCR was performed as described previously [13] except that qPCR primers named AMP were used (Additional file 1: Table S1). TPI1 qPCR primers were used as internal standard.

### Whole-cell transamination

50 ml sealed serum flasks with magnetic stirring (140 rpm) and 5 or 25 g/l cell dry weight (dw) at 30 °C were used for whole-cell transamination. The reaction solution contained 10 ml 100 mM sodium phosphate buffer (pH 7.0), 100 g/l glucose, 25 mM *racemic* 1-PEA, and 0–0.1 mM PLP. For direct asymmetric transamination of acetophenone to (*S*)-1-PEA, the reaction solution contained the same buffer, 25 g/l cell dry weight (dw), 500 mM l-alanine, 0.1 mM PLP, 50 g/L glucose and 10 mM acetophenone.

### Analyses

Growth was monitored by measuring optical density at a wavelength of 620 nm (OD_620_) with an Ultrospec 2100pro spectrophotometer (Amersham Biosciences, Sweden). Cell dry weight determination was performed as previously described, as was the determination by normal and reverse phase HPLC of (*R*)-1-phenylethylamine, (*S*)-1-phenylethylamine, (*R*)-1-phenylethanol, (*S*)-1-phenylethanol, acetophenone, glucose, glycerol, acetate, succinate, and ethanol [12]. Pyruvate was detected by the same HPLC method as previously described for glucose and its metabolites [12], except that a UV spectrophotometric detector (Shimadzu SPD-6A) at 214 nm was used instead.

## Additional file

**Additional file 1.** Additional material.
