# Supplementary material for: Improvement of whole-cell transamination with Saccharomyces cerevisiae using metabolic engineering and cell pre-adaptation
Source: Microb Cell Fact. 2017 Jan 3;16:3. doi: 10.1186/s12934-016-0615-3 (PMC5209827; doi:10.1186/s12934-016-0615-3)
Supplement: Supplementary file 1 — Additional file 1. Additional material. [file 12934_2016_615_MOESM1_ESM.docx]

Additional material

**Improvement of whole-cell transamination with *Saccharomyces cerevisiae* using metabolic engineering and cell pre-adaptation**

Nora Weber^1,2^, Marie Gorwa-Grauslund^1^, and Magnus Carlquist^1,^*

**NW:** noraw@evolva.com, ^1^Division of Applied Microbiology, Department of Chemistry, Faculty of Engineering, Lund University, PO Box 124, SE-221 00 Lund, Sweden.

Present address: ^2^ Evolva SA, Duggingerstrasse 23, CH-4153 Reinach, Switzerland.

**MGG:** marie-francoise.gorwa@tmb.lth.se, ^1^Division of Applied Microbiology, Department of Chemistry, Faculty of Engineering, Lund University, PO Box 124, SE-221 00 Lund, Sweden.

**MC:** magnus.carlquist@tmb.lth.se, ^1^Division of Applied Microbiology, Department of Chemistry, Faculty of Engineering, Lund University, PO Box 124, SE-221 00 Lund, Sweden.

^*^ Corresponding author. Phone: +46 46 222 9875, Email: magnus.carlquist@tmb.lth.se

**Supplementary material**

**Table S1.** Primers used in this study.

**Figure S1.** Cultivation of CV-TA TAM (TMB4374) and control strain (TMB4369) in shake flasks with defined mineral medium.

**Figure S2.** HPLC chromatograms for asymmetric synthesis of (*S*)-1-phenylethylamine from acetophenone.

**Figure S3.** Conversion of *racemic* 1-phenylethylamine with cell extract from yeast strains expressing three different recombinant ω-transaminases.

Table S1. Primers used in this study.

| **Name** | **Sequence 5’→3’** |
| --- | --- |
| CV fwd | TTCGACGGATTCTAGATGCAAAAACAAAG |
| CV rev | AGTCCAAAGCTCTAGTATGCTAAACCTCT |
| OA fwd | TTCGACGGATTCTAGATGACAGCACAA |
| OA rev | AGTCCAAAGCTCTAGTTATCTAGTTAAAG |
| AMP qPCR fwd | CAGTGCTGCAATGATACCGC |
| AMP qPCR rev | GTGACACCACGATGCCTGTA |
| TPI1 qPCR fwd | TCAGGTTGGTGGAAGATTAC |
| TPI1 qPCR rev | GCCCTTTATATTCCCTGTTAC |

**a)
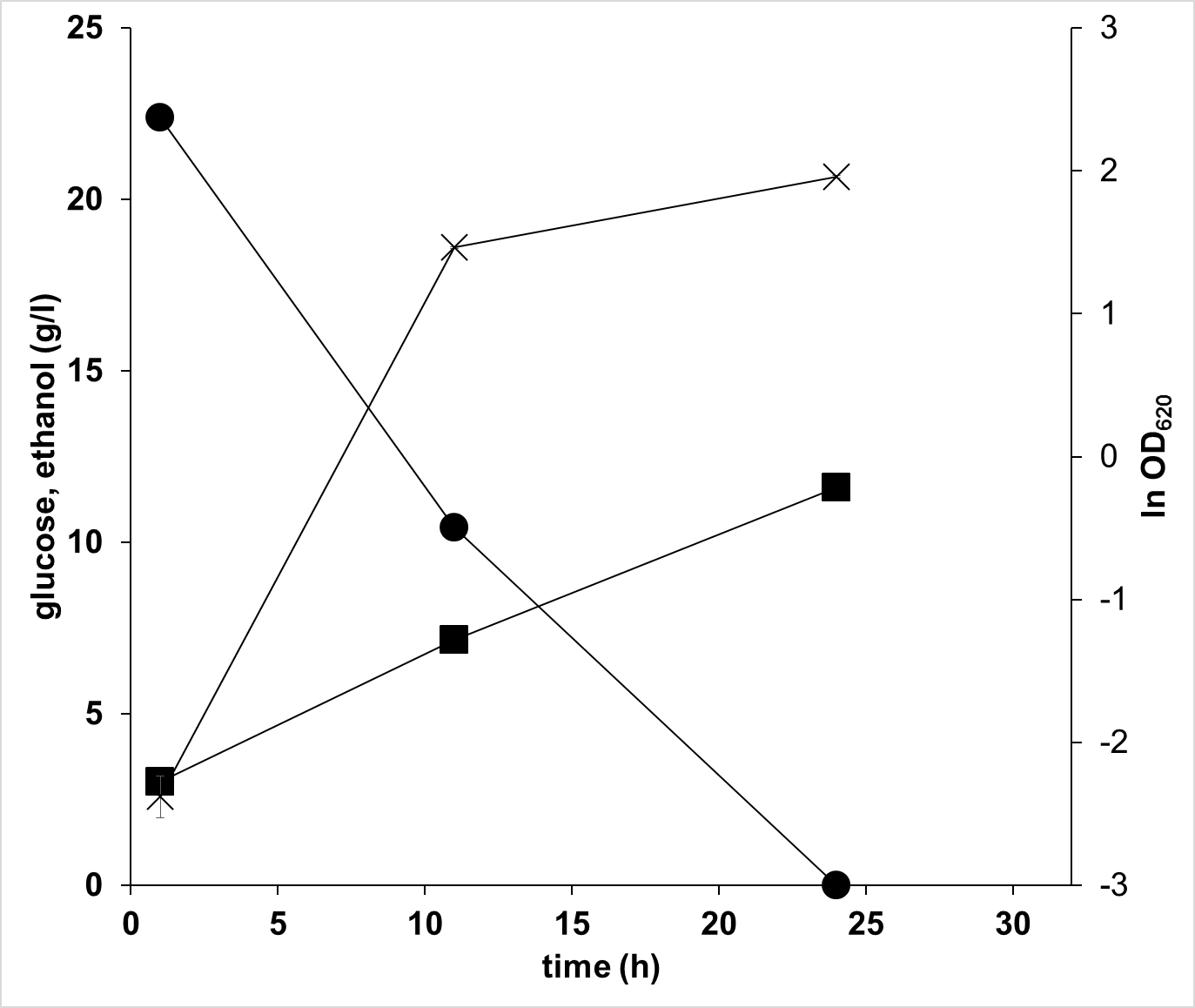
**

**b)
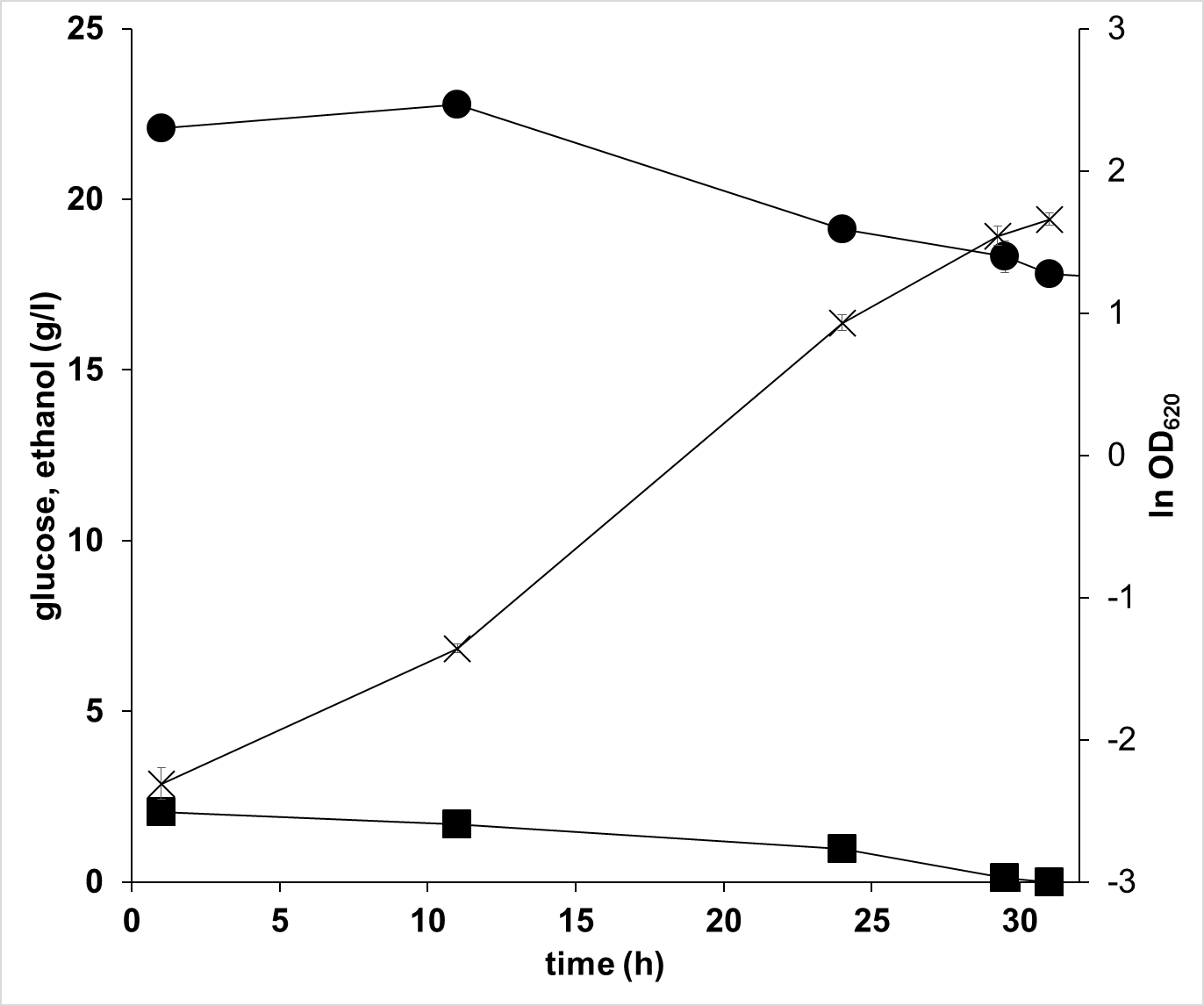
**

Figure S1. Growth and metabolite production for TMB4369 (CV-TA) (a) and TMB4374 (CV-TA TAM) (b) strain in mineral medium [1] with 20 g/l glucose and 3 g/l ethanol inoculated at OD_620_ 0.1. Glucose (circle), ethanol (square), ln OD (cross).

**
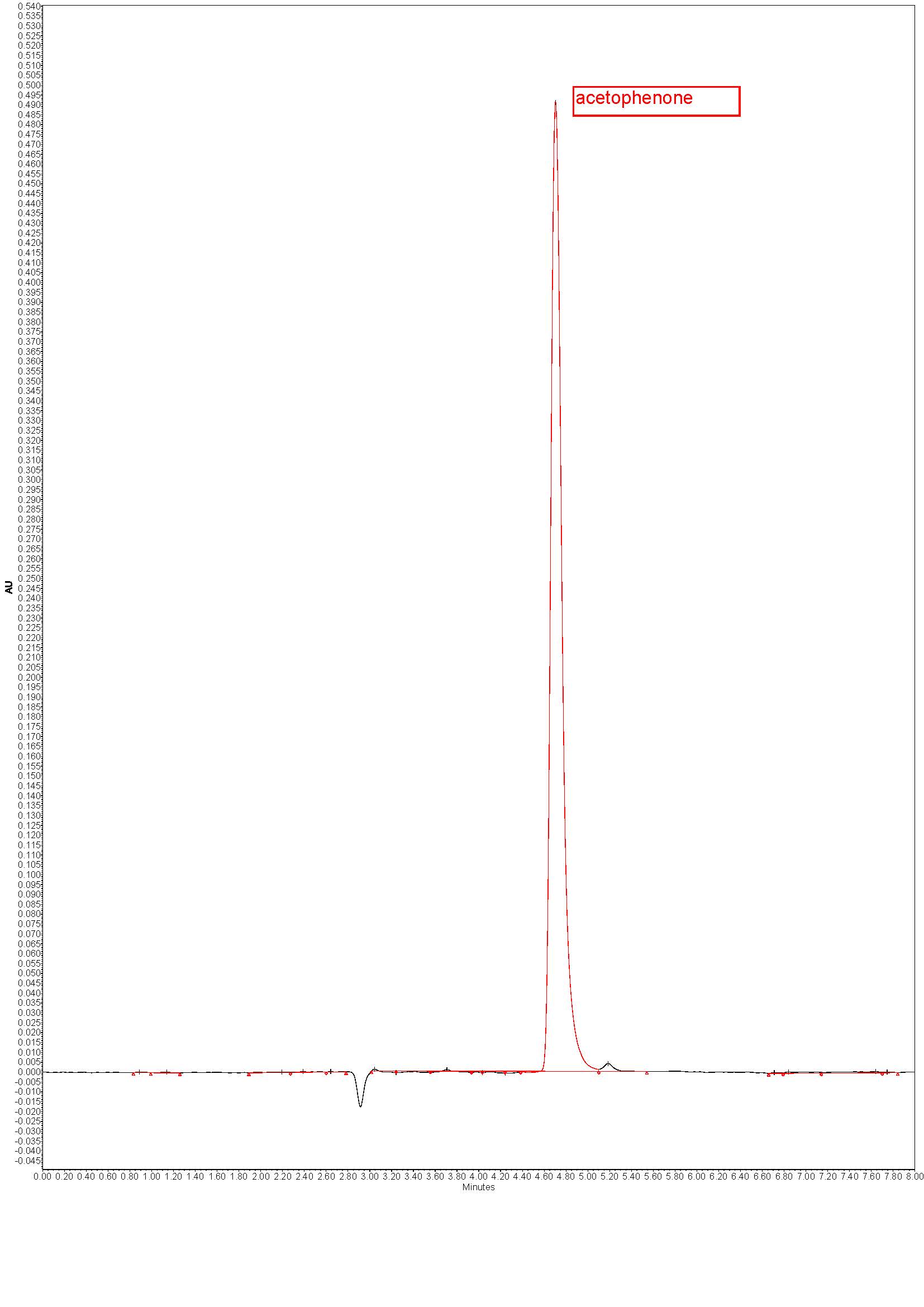
**

**a)**

**
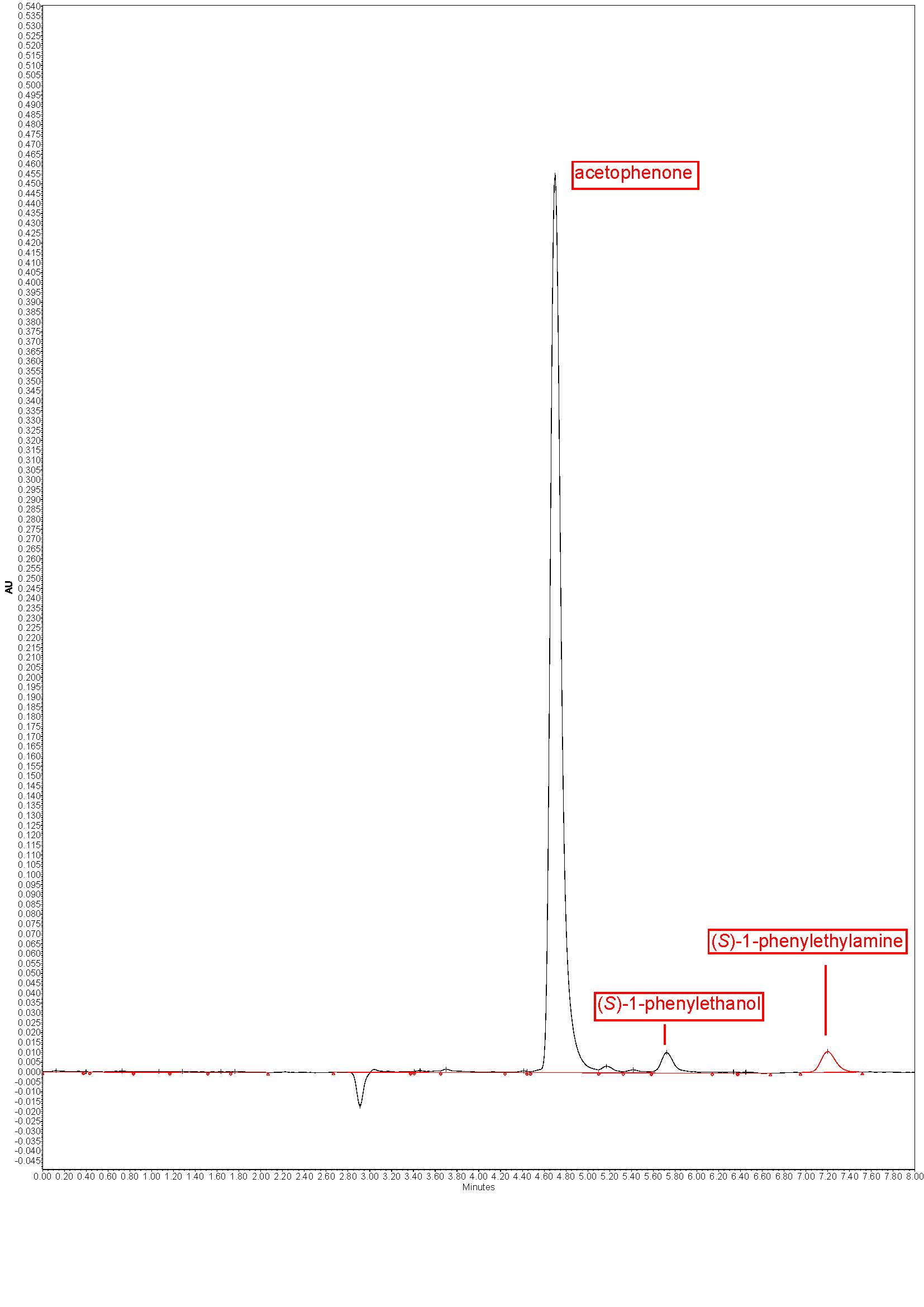
**

**b)**

**Figure S3. Whole-cell asymmetric synthesis of (*S*)-1-phenylethylamine from acetophenone with TMB4375 containing 6 copies of CV-TA**. **a**) HPLC chromatogram from time point t=0 h; **b**) HPLC chromatogram from time point t=24 h. Experiments were performed in biological triplicates using 10 mM acetophenone, 50 g/l glucose, 500 mM L-alanine, and whole-cells (5 g/L cdw) in defined mineral media [1] at pH 6.5.


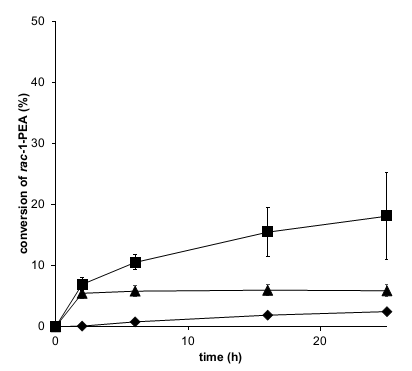


**Figure S2.** Kinetic resolution of *racemic* 1-phenylethylamine (PEA) with cell extract of *S. cerevisiae* strains TMB4367, TMB4369 and TMB4371 containing CC-TA (diamond), CV-TA (triangle), and OA-TA (square), respectively.

Experiments were performed in biological duplicates using 100 mM (8.8 g/l) pyruvate, 50 mM *racemic* 1-PEA, 0.1 mM PLP, and cell extract (2 mg/ml total protein). The conversion (%) refers to *racemic* 1-PEA, with a theoretical maximum of 50%. CC-TA, CV-TA, and OA-TA strains were grown as described in the materials and methods section of the article. Cell extract was prepared with glass beads (0.5 mm), 100 mM phosphate buffer pH 7.0, and precellys 24 bead beater with a cryolys cooling unit (Bertin technologies, Aix-en-Provence Cedex, France). Total protein amount was determined by Bradford with bovine serum albumin as standard [2].

**References**

[1] Verduyn C, Postma E, Scheffers W *et al.* Effect of benzoic acid on metabolic fluxes in yeasts: a continuous-culture study on the regulation of respiration and alcoholic fermentation. *Yeast* 1992;**8**: 501-17.

[2] Bradford MM. A rapid and sensitive method for the quantitation of microgram quantities of protein utilizing the principle of protein-dye binding. *Anal Biochem* 1976;**72**: 248-54.
